# Depletion of Arginine by Recombinant Arginine Deiminase Induces nNOS-Activated Neurotoxicity in Neuroblastoma Cells

**DOI:** 10.1155/2014/589424

**Published:** 2014-07-14

**Authors:** Shan-Erh Lin, Fe-Lin Lin Wu, Ming-Feng Wei, Li-Jiuan Shen

**Affiliations:** ^1^School of Pharmacy, College of Medicine, National Taiwan University, Taipei 10050, Taiwan; ^2^Graduate Institute of Clinical Pharmacy, Medical College, National Taiwan University, Taipei 10050, Taiwan; ^3^Department of Pharmacy, National Taiwan University Hospital, Taipei 10048, Taiwan

## Abstract

The abnormal regulation of inducible nitric oxide synthase (iNOS) and neuronal nitric oxide synthase (nNOS) is associated with neurodegenerative disorders. Recombinant arginine deiminase (rADI) is a selective NO modulator of iNOS and eNOS in endothelial cells, and it also exhibits neuroprotective activity in an iNOS-induced neuron-microglia coculture system. However, the effect of rADI on nNOS remains unknown. Addressing this issue is important for evaluating the potential application of rADI in neurodegenerative diseases. SH-SY5Y cells were treated with *N*-methyl-D-aspartic acid (NMDA) to activate nNOS. NMDA increased NO production by 39.7 ± 3.9% via nNOS under arginine-containing conditions, but there was no significant increase in both arginine-free and rADI pretreated arginine-containing (citrulline) buffer. Subsequently, neither NMDA nor rADI alone caused cytotoxicity, whereas cotreatment with NMDA and rADI resulted in dissipation of the cell mitochondrial membrane potential and decreased cell viability. The mechanism of rADI cytotoxicity in the presence of NMDA is caused by the inhibition of NO production via nNOS mediated by the NMDA receptor, which was abolished when extracellular arginine was absent, even in the presence of citrulline. rADI not only reduced NO production but also caused cellular toxicity in nNOS-activated SH-SY5Y cells, suggesting a dual role for rADI in NOS-mediated neurotoxicity.

## 1. Introduction

Nitric oxide (NO) is important for many synthases (NOSs, EC 1.14.13.39), including inducible nitric oxide synthase (iNOS), neuronal nitric oxide synthase (nNOS), and endothelial nitric oxide synthase (eNOS) [1]. The distribution and physiological functions of these different isoforms vary [2]. In the central nervous system (CNS), nNOS is abundant in neurons and astrocytes [3]. iNOS and eNOS are found in glial cells, endothelial cells, and astrocytes [4, 5]. Evidence indicates that abnormal regulation of iNOS and nNOS is associated with neurodegenerative disorders, such as cerebral ischemia [6–8]. In the CNS, glial cells express iNOS, which exhibits no significant effects under physiological conditions but is induced in response to immunological stimuli, producing NO that exerts immunological functions [9]. Neurons and astrocytes express nNOS, a constitutively expressed protein that is activated when glutamate binds to* N*-methyl-D-aspartate (NMDA) receptors during long-term potentiation [10]. Therefore, the selectivity of an NO modulator on NO isoforms is important for its effect on the physiological or pathophysiological functions of the CNS.

Arginine deiminase (ADI, EC 3.5.3.6) catalyzes the hydrolysis of arginine to citrulline and ammonia and was first discovered in* Mycoplasma arginini*. Arginine is an amino acid that is involved in protein synthesis and is also the sole substrate of NOSs. Recombinant arginine deiminase (rADI) has been developed as an anticancer drug for patients with melanoma and hepatocellular carcinoma because of its antitumor and antiangiogenesis activities* in vitro* and* in vivo* [11]. In addition, rADI is a selective NO modulator of iNOS and eNOS [12]. rADI selectively inhibits NO produced via iNOS rather than eNOS in endothelial cells, which might be because of the accessibility of NOSs to the citrulline-arginine regeneration compartment [13]. Therefore, rADI is an appropriate selective NO modulator of iNOS-mediated adverse processes such as inflammation and sepsis [14].

We previously used a coculture system of neurons and microglia to demonstrate the protective effect of rADI on iNOS-mediated neurotoxicity [15]. rADI retained LPS (lipopolysaccharide) and interferon-*γ* stimulated neuronal viability and also preserved neuronal function in the coculture system. nNOS activation plays a significant role in excitotoxic neuronal death [16–18]. However, the effect of rADI on NO-related neurodegenerative disorders is yet to be determined because what extent rADI affects NO production via nNOS remains unknown. Therefore, in the present study, the effect of rADI on nNOS-activated neurons was further elucidated in SH-SY5Y neuroblastoma cells. Our findings provide important information regarding the depletion of arginine by rADI in NO-mediated neuronal diseases.

## 2. Materials and Methods

### 2.1. Materials

The ADI plasmid of* Mycoplasma arginini* was a kind gift from Dr. Wei-Chiang Shen (School of Pharmacy, University of Southern California, Los Angeles, USA) and was transformed into* Escherichia coli* (*E. coli*) BL21. rADI was prepared and purified to homogeneity as previously described [19]. The reagents for the* E. coli* culture medium were purchased from Becton Dickinson Transduction Laboratory (Lexington, KY). Ampicillin was purchased from MDBio (Rockville, Maryland). The Micro BCA protein assay reagent kit was obtained from Pierce (Rockford, IL). Isopropyl *β*-D-1-thiogalactopyranoside (IPTG), dithiothreitol (DTT), 3-(4,5-dimethylthiazol-2-yl)-2,5-diphenyl tetrazolium bromide (MTT), sulfanilamide, N-(1-napthyl)-ethylenediamine-2HCl, retinoic acid (RA), 2,3-diaminonaphthalene (DAN),* N*-methyl-D-aspartic acid (NMDA), (+)-MK-801, and rhodamine 123 (Rho 123) were purchased from Sigma (St. Louis, MO).

### 2.2. Cell Culture

All cell culture reagents were purchased from Gibco (Grand Island, NY), and all cells were incubated in a humidified atmosphere with 5% CO_2_ at 37°C. Human neuroblastoma SH-SY5Y cells were a kind gift from Dr. Fan-Lu Kung (School of Pharmacy, National Taiwan University, Taipei, Taiwan).

The SH-SY5Y cells were maintained in a mixture of F12 nutrient mixture (Ham) and minimum essential medium (MEM) in a 1 : 1 ratio containing 10% heat-inactivated fetal bovine serum (FBS), 50 U/mL penicillin, and 50 *μ*g/mL streptomycin. The medium was renewed every other day.

### 2.3. Activation of nNOS in SH-SY5Y Cells

SH-SY5Y cells were seeded at a density of 8 × 10^4^ in 6-well plates with an equal volume of MEM and F12 enriched with 10% FBS. After 24 h, the serum in the medium was reduced to 1%, and 10 *μ*M retinoic acid (RA) was added for cell differentiation. At day 7, the cells formed a delicate dendritic network and were ready for the experiments.* N*-methyl-D-aspartic acid (NMDA; 1 mM) was used to activate nNOS.

### 2.4. NO Measurement

NO was measured using the DAN method, with a detection limit of 10 nM [20]. The method was utilized to quantify NO produced in the cell culture in which nNOS produces relatively low amounts of NO. The DAN method is a fluorometric method for nitrite quantification. Nitrite reacts with DAN, a nonfluorescent compound, under acidic conditions to form 2,3-diaminonaphthotriazole, a highly fluorescent compound. This assay was performed as previously described [20]. The supernatant of cell culture was centrifuged at 1000 rpm for 5 min and reacted with 100 *μ*L of 50 ng/mL DAN in 0.62 N HCl for 15 min. The reaction was terminated by adding 50 *μ*L of 2.8 N NaOH. The amount of NO was determined according to the fluorescence intensity of the azole fluorophore using a Hitachi F-4500 fluorescence spectrophotometer (Tokyo, Japan), with excitation at 365 ± 10 nm and emission at 450 ± 10 nm.

To determine whether NO is specifically produced via nNOS, vinyl-L-NIO and 1400W were used. Vinyl-L-NIO is a selective nNOS inhibitor, with Ki values of 0.1, 12.0, and 60 *μ*M for nNOS, eNOS, and iNOS, respectively [21]. 1400W is a selective iNOS inhibitor, with IC50 values of 0.23, 7.3, and 1000 *μ*M for iNOS, nNOS, and eNOS, respectively [22]. The cells were pretreated with 2 *μ*M vinyl-L-NIO and 1 *μ*M 1400W 24 h prior to the experiments. Differentiated SH-SY5Y cells were washed twice with Mg^2+^-free Krebs-Henseleit buffer (120 mM NaCl, 2 mM KCl, 26 mM NaHCO_3_, 1.18 mM KH_2_PO_4_, 11 mM glucose, and 2 mM CaCl_2_, pH 7.4) and treated with control or 1 mM NMDA in the continuous presence of NOS inhibitors in Mg^2+^-free Krebs-Henseleit buffer containing 30 *μ*M glycine and 1 mM arginine for 1 h. Then, the buffer was collected for NO measurement using the DAN method.

To understand the effect of arginine deprivation on NO production, Mg^2+^-free Krebs-Henseleit buffer containing 30 *μ*M glycine with or without 1 mM arginine or with 1 mM arginine plus 1 mU/mL rADI was prepared 24 h prior to the experiments and stored at 37°C. The cells were washed and incubated with control or NMDA in the above buffer for 1 h, after which, the buffer was collected for NO measurement using the DAN method.

### 2.5. Analysis of Arginine Concentration in rADI Pretreated Buffer

In order to demonstrate the enzymatic activity of rADI, concentrations of arginine and citrulline in the buffer of arginine + rADI pretreatment were analysed by ACQUITY UPLC (Waters, Milford, MA, USA). An AccQ-Tag Ultra Derivatization Kit (Waters, Milford, MA, USA) was used for the derivatization of arginine and citrulline, and the derived compounds were further quantified by UPLC.

### 2.6. Measurement of Mitochondrial Membrane Potential

Dissipation of the mitochondrial membrane potential is an indicator of early apoptosis and is also a key event in excitotoxicity [23]. Rhodamine 123, a cationic dye that incorporates into mitochondria based on the membrane potential, was used to measure the mitochondrial membrane potential. The medium was pretreated with 1 mU/mL rADI 24 h prior to the experiments and stored at 37°C. At the beginning of the experiment, the cells were washed twice with Mg^2+^-free Krebs-Henseleit buffer. Then, the cells were treated with control or 1 mM NMDA in normal medium, rADI-pretreated medium, 1 mM NMDA in rADI-pretreated medium, or 1 mM NMDA in rADI-pretreated medium replenished with 0.8 mM L-arginine for 24 h. The cells were incubated with 5 *μ*M rhodamine 123 in the dark for 40 min. Then, the cells were harvested and their fluorescence intensity was measured using a BD FACSCalibur flow cytometer (Franklin Lakes, NJ). Rhodamine 123 was excited using the 488 nm argon laser and its emission was detected in the FL1 channel. The data were analyzed using the BD CellQuest 4.2 software (Franklin Lakes, NJ) and are presented as histogram plots using logarithmic scales.

### 2.7. Cell Viability Assay

The effect on cell viability was evaluated using the MTT (3-[4,5-dimethylthiazol-2-yl]-2,5-diphenyl tetrazolium bromide) assay [24]. Differentiated cells were prepared as previously described. Medium with 1% FBS was pretreated with rADI (1 mU/mL) 24 h prior to the experiment to deplete the arginine in the medium. All rADI treatments in this experiment represent cells that were cultured in the rADI pretreated medium. The replenishment of 0.8 mM arginine to the rADI pretreated medium at the beginning of this experiment mimicked the amount of arginine in the standard medium. The cells were incubated with control, 1 mM NMDA, 1 mU/mL rADI, a combination of NMDA and rADI, or the combination of NMDA and rADI with arginine replenishment for 24 h. To test the effect of an nNOS inhibitor and NMDA receptor antagonist on cell viability under nNOS activation, 5 *μ*M vinyl-L-NIO and 0.2 *μ*M (+)-MK-801 were administered 24 h and 1 h before NMDA treatment, respectively. After 24 h of NMDA treatment, cell viability was determined using the MTT assay. The cells were incubated with 1 mg/mL MTT for 2 h. DMSO was added to solubilize the formazan crystals, and the absorbance at 560 nm was measured with a BioTek Power Wave XS plate reader.

### 2.8. Data Analysis

The data are presented as the mean ± SEM. Statistical analysis was performed with the SPSS program. The differences among the groups were evaluated using either a* t*-test or ANOVA, followed by Bonferroni's modified *t*-test. A *P* value less than 0.05 indicates statistical significance.

## 3. Results

### 3.1. nNOS Activation in a Neuroblastoma Culture

**Figure 1:**
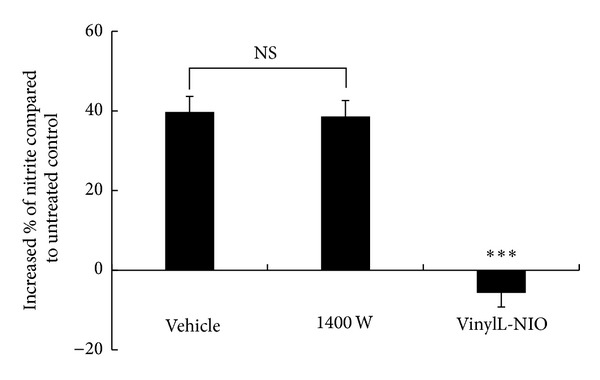
NMDA-activated NO production in differentiated SH-SY5Y cells. SH-SY5Y cells were seeded in 6-well plates and differentiated for 6 days. The cells were pretreated with vehicle, 1 *μ*M 1400W, or 2 *μ*M vinyl-L-NIO for 24 h. The cells were washed with Mg^2+^-free Krebs-Henseleit buffer twice followed by exposure to untreated control or 1 mM NMDA with 1 mM arginine and 30 *μ*M glycine in Mg^2+^-free Krebs-Henseleit buffer with the continuous presence of 1400W or vinyl-L-NIO for 1 h. The supernatant was collected for NO measurement using the DAN method. The data are the mean ± SEM (*n* = 9 in each group). ***P < 0.001 versus vehicle; NS: no significant difference.

nNOS activity was successfully induced by NMDA in differentiated SH-SY5Y cells treated with 10 *μ*M retinoic acid (RA) for 7 days. NO production via nNOS in the presence of NMDA was increased by 39.7 ± 3.9% over the control in the absence of NMDA (Figure 1). The concentrations of NO were 109.9 ± 8.8 nM and 150.7 ± 8.7 nM in the absence and presence of NMDA, respectively.

SH-SY5Y cells were further pretreated with selective NOS inhibitors to confirm that NMDA-induced NO was produced via nNOS. When the cells were pretreated with 1 *μ*M 1400W, a selective iNOS inhibitor, 38.6 ± 4.1% of NMDA-activated NO production still remained, which was not significantly different from the vehicle-treated group. However, when the cells were pretreated with 2 *μ*M vinyl-L-NIO, a selective nNOS inhibitor, no significant NO induction by NMDA was observed (Figure 1). Taken together, we conclude that NMDA-activated NO was produced via nNOS and not iNOS.

### 3.2. Effect of Arginine Deprivation on nNOS-Activated NO Production

**Figure 2:**
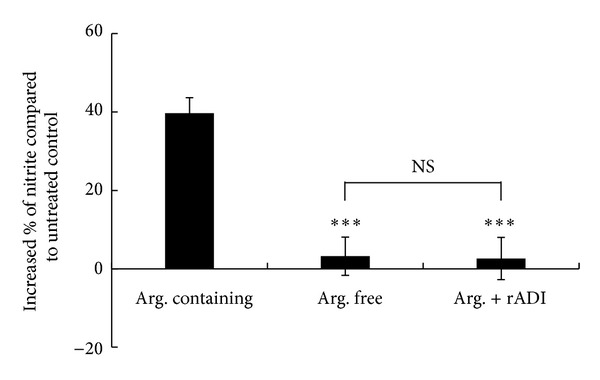
Effect of rADI on nNOS-activated NO production in differentiated SH-SY5Y cells. Different buffers (1 mM arginine, arginine-free, or 1 mM arginine plus 1 mU/mL rADI) were prepared in magnesium-free Krebs-Henseleit buffer with 30 *μ*M glycine 24 h prior to the experiments and incubated at 37°C. The differentiated cells were washed with Mg^2+^-free Krebs-Henseleit buffer twice followed by exposure to untreated control or 1 mM NMDA in the prepared buffers, respectively, for 1 h. The supernatant was collected for NO measurement using the DAN method. The data are the mean ± SEM (*n* = 12 in each group). ***P < 0.001 versus arginine-containing buffer; arg: arginine; NS: no significant difference.

After the establishment of the nNOS-activated cell culture, we investigated the effect of arginine deprivation on NMDA-activated NO production in the cells. The effect of rADI on arginine deprivation was confirmed by UPLC. In rADI pretreated arginine-containing buffer, the level of arginine was reduced from 1.00  ±  0.03 mM to 0.04  ±  0.01 mM after the 24 h incubation with rADI. Correspondently, the concentration of citrulline was increased from undetectable to 0.98 ± 0.02 mM. The cells in arginine-containing buffer, arginine-free buffer, and rADI pretreated arginine-containing buffer were treated with NMDA for 1 h. The results in Figure 2 show that NMDA-activated NO production was reduced from 39.7 ± 3.9% in arginine-containing buffer to 3.2 ± 4.9% and 2.6 ± 5.4% in arginine-free buffer and rADI pretreated arginine-containing buffer, respectively, indicating that NO production via nNOS is dependent on the extracellular arginine in SH-SY5Y cells. The extracellular citrulline in the rADI pretreated arginine-containing buffer could not be used as the substrate for nNOS.

### 3.3. Effect of rADI SH-SY5Y Survival upon NMDA Activation

**Figure 3:**
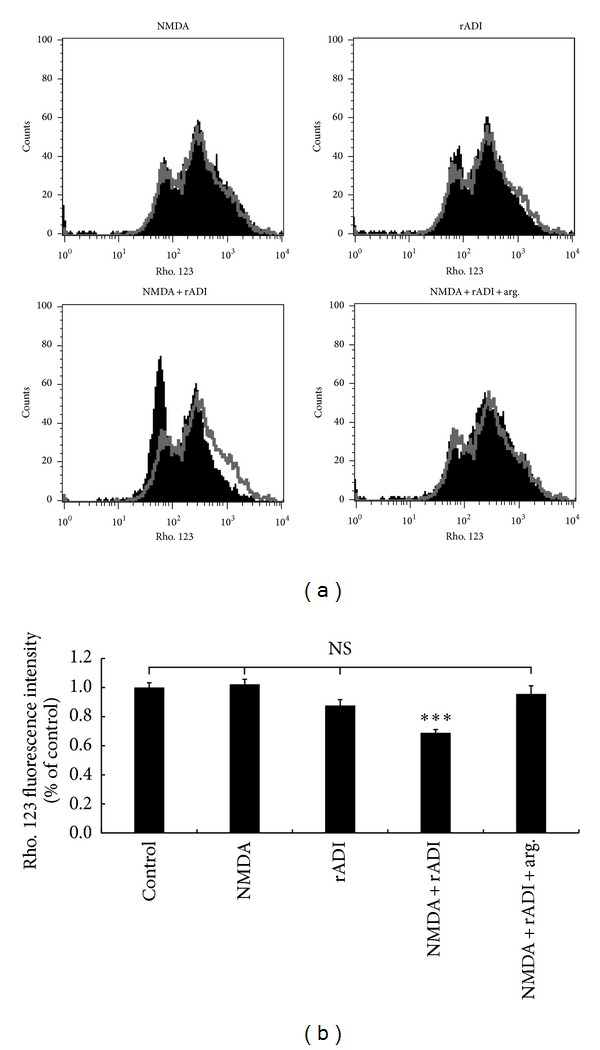
Effect of rADI on mitochondrial membrane potential in SH-SY5Y cells. The differentiated cells were washed twice with Mg^2+^-free Krebs-Henseleit buffer and treated with control, 1 mM NMDA in normal medium, rADI pretreated medium, 1 mM NMDA in rADI pretreated medium, or 1 mM NMDA in rADI pretreated medium replenished with 0.8 mM arginine for 24 h. The cells were incubated with 5 *μ*M rhodamine 123 in the dark for 40 min. The mitochondrial membrane potential was measured by flow cytometry. (a) Representative flow charts. The black peaks represent the rhodamine fluorescence intensity of the indicated treatments, and the gray peaks represent the control. (b) The quantitative rhodamine fluorescence intensity presented as percentage of the control. The data are the mean ± SEM (*n* = 9) ***P < 0.001 versus control; arg: arginine.

**Figure 4:**
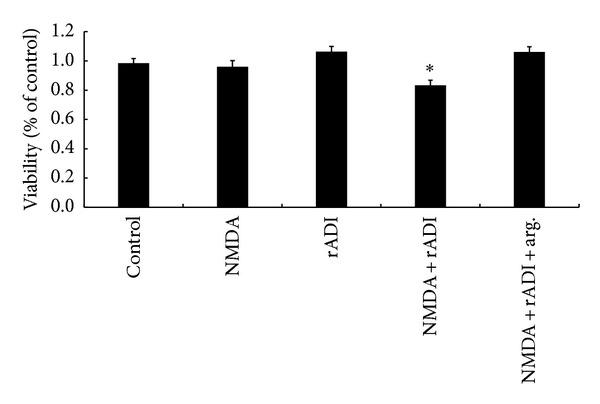
Effect of rADI on cell viability in NMDA-treated SH-SY5Y cells. The differentiated cells were incubated with control, 1 mM NMDA, 1 mU/mL rADI, cotreatment with NMDA and rADI, or cotreatment with arginine replenishment for 24 h. Cell viability was measured using the MTT assay. The error bars represent the SEM (*n* = 12). *P < 0.05 versus control; arg: arginine.

To understand the effect of rADI on neuroblastoma cell survival upon NMDA activation, both the mitochondrial membrane potential and MTT assay were used to evaluate cell viability.  Figure 3 shows the results of the rhodamine 123 fluorescence intensity measured in cells under the following conditions: control without any treatment, 1 mM NMDA in normal medium, absence of NMDA in rADI pretreated medium, 1 mM NMDA in rADI pretreated medium, and 1 mM NMDA in rADI-pretreated medium replenished with 0.8 mM arginine for 24 h. According to the flow chart and quantitative analysis of rhodamine 123 fluorescence shown in Figure 3, neither NMDA treatment nor rADI pretreated medium resulted in the dissipation of the mitochondrial membrane potential. However, when the cells were exposed to NMDA in rADI pretreated medium, the mitochondrial membrane potential depolarized to 68.8 ± 2.4% of the control group, which was statistically significant (*P* < 0.001). This disruption of the mitochondrial membrane potential was restored to 95.6 ± 5.6% by replenishing the arginine at the beginning of the NMDA treatment under the rADI pretreatment condition. Similar results were obtained using the MTT assay (Figure 4). The cellular viability was significantly reduced (*P* < 0.05) in the NMDA and rADI pretreated cells only. Therefore, the NMDA activated the nNOS but did not cause excitotoxicity in our* in vitro* model. However, rADI had an excitotoxic effect on nNOS-activated neuroblastoma cells.

(+)-MK-801 was used as an NMDA receptor antagonist at 0.2 *μ*M, which effectively suppresses nNOS activity [25]. (+)-MK-801 blocked the toxicity of rADI upon NMDA treatment (Table 1). The cell viability was increased to 96.3 ± 1.0% from 87.4 ± 1.6% after (+)-MK-801 was administered to the groups treated with rADI and NMDA. In addition, the combination of vinyl-L-NIO and NMDA reduced the cell viability to 86.7 ± 1.2% of the control, but vinyl-L-NIO alone did not (Table 2).

## 4. Discussion

In this study, we found that rADI was deleterious to nNOS-activated neuroblastoma cell cultures; however, it was reported as beneficial to iNOS-mediated neurotoxicity in a neuronal and microglial coculture [15].

The neuroblastoma cell line SH-SY5Y is the thrice cloned subline of SK-N-SH cells [26]. SK-N-SH cells express functional NMDA receptors when differentiated by RA [27]. NMDA and glutamate increased the intracellular Ca^2+^ levels in differentiated SK-N-SH cells but not in undifferentiated cells [27]. RA (10 *μ*M) was used to differentiate the SH-SY5Y cells, and an increase in NO production of approximately 40% via nNOS was observed in our study (Figure 1). The NO production via nNOS but not iNOS was further confirmed using selective NOS inhibitors (Figure 2). Therefore, differentiated SH-SY5Y cells treated with RA may be an appropriate model for nNOS studies.

In our neuroblastoma cell culture model, no neurotoxicity was elicited by NMDA when the cells were incubated in arginine-containing medium, indicating that the differentiated SH-SY5Y cells were resistant to nNOS activation (Figures 3 and 4). These results are consistent with a previous report that some neurons are resistant to nNOS-mediated neurotoxicity [28]. By contrast, excitotoxic neuronal apoptosis caused by the activation of NMDA receptors has been reported [18, 29]. Knockout mouse experiments also showed an adverse role for nNOS during hypoxic-ischemic injury [6, 30]. Despite evidence demonstrating the detrimental role of nNOS, other studies have shown that the inhibition of NO formation was not beneficial for NMDA toxicity in a murine cortical culture system [31].

Although NMDA was not harmful to the SH-SY5Y cells in normal medium in our cell culture model, toxicity was evoked in the arginine-depleted medium, and replenishing the arginine prevented the neurotoxicity (Figures 3 and 4). This finding is consistent with studies suggesting that arginine plays a protective role. Grima et al. treated cortical neuronal cultures with NMDA and found that, in the absence of arginine, NMDA induced a decrease in the neuronal mitochondrial membrane potential and cell death, and this outcome was reversed by the addition of arginine [32]. A similar result was observed in nNOS-transfected human kidney 293 cells in which NO generation was triggered by A23187, a calcium ionophore. A23187 did not cause cell injury in normal medium but significantly induced LDH release in arginine-free medium [33]. One possible reason for the protective effect of arginine may be the scavenging and elimination of superoxide, which is generated when arginine availability decreases [32–34]. Superoxide reacts with NO to form the particularly toxic peroxynitrite (ONOO^−^), which interrupts mitochondrial function and leads to dissipation of the membrane potential. In addition, we demonstrated that the NMDA antagonist (+)-MK-801 prevented cell toxicity when the cells were incubated with rADI and NMDA (Table 1), indicating that the cytotoxicity of the combination of rADI and NMDA was mediated by the activation of NMDA receptors. We further found that the nNOS inhibitor vinyl-L-NIO is toxic to cells, similar to rADI in cells treated with NMDA (Table 2). Based on these results, we conclude that rADI is toxic to cells upon NMDA treatment through the inhibition of NO via nNOS. Apoptosis has been reported in a NMDA excitotoxic cell death in isolated chick embryo retia model [35]. It can be further investigated whether the cells undergo apoptosis or necrosis upon the stimulation with rADI and NMDA. By contrast, it has previously been shown that the arginine deprivation was beneficial to nNOS-mediated toxicity. Dawson et al. demonstrated that, in primary cortical cultures, NO mediated both glutamate and NMDA neurotoxicity and was prevented by NOS inhibitors [17]. They also showed that NMDA-evoked toxicity was attenuated by the depletion of arginine in the medium, either via arginase pretreatment or arginine-free medium.

Matsuoka et al. demonstrated that the microinjection of LPS/IFN-*γ* into rat hippocampus caused neuronal apoptosis, which was mediated by microglial activation and iNOS induction [36]. Cunningham et al. also showed that central and systemic LPS challenge induced neuronal apoptosis [37]. In the coculture model, we previously demonstrated that rADI reduced iNOS-mediated neurotoxicity and that the neuroprotective effect of rADI was abolished by arginine replenishment [15]. Arginine deprivation by rADI was beneficial for alleviating iNOS-mediated neurotoxicity in our previous study [15] but was harmful to nNOS-activated SH-SY5Y cells in this study. Because nNOS and iNOS play important roles during the early and late stages of ischemia, respectively, the role of arginine can be understood using the ischemic model. Administration of arginine within 20 min following ischemia ameliorated cerebral blood flow and reduced infarct volume [38, 39], whereas arginine resulted in a worse outcome when administered 12 h following middle cerebral artery occlusion (MACO) [40]. These results indicate that arginine is beneficial during the early ischemic stage but detrimental during the late ischemic stage, consistent with our findings in the cell culture model.

rADI selectively inhibits iNOS-induced NO production rather than eNOS-mediated NO production [12]. A possible reason for this is that eNOS utilizes intracellularly regenerated arginine from citrulline [13]. Therefore, the depletion of extracellular arginine by rADI did not affect eNOS-mediated NO production. However, iNOS uses extracellular arginine as the only substrate [13]. Therefore, rADI inhibits iNOS-induced NO production and protects cells from iNOS-mediated toxicity [15]. In our cell culture system, NMDA-activated NO production was dependent upon extracellular arginine, but not citrulline (Figure 2). This finding can be explained by the lack of argininosuccinate synthetase (AS), a rate-limiting enzyme for arginine-citrulline regeneration in SH-SY5Y cells (data not shown). Therefore, SH-SY5Y cells could not convert citrulline to the NO precursor arginine. In the present study, we did not determine whether AS-positive neurons use citrulline as an nNOS substrate to generate NO. nNOS colocalizes with the arginine regeneration enzymes [41–44] and uses citrulline as a substrate in the rat gastric fundus [45]. Regeneration of arginine from citrulline has also been identified in the perivascular nerves of cerebral arteries [46], where nNOS is widely expressed [47]. Therefore, nNOS is likely to use citrulline to synthesize NO, but this must be further investigated.

## 5. Conclusion

In summary, we demonstrated that rADI protects cells from iNOS-mediated toxicity but impairs nNOS-activated cells. This study indicates that rADI has deleterious effects on nNOS-activated neurons when used to treat iNOS-mediated neuronal disorders. Further studies are necessary to carefully elucidate the treatment paradigm for rADI in neuronal disorders, and the timing and dose of rADI should also be considered.

## Figures and Tables

**Table 1:** The NMDA antagonist (+)-MK-801 prevents the rADI cytotoxicity upon nNOS-activation.

Cell viability	Control	rADI + NMDA	rADI + NMDA + (+)-MK-801
% of control	100 ± 2.8	87.4 ± 1.6∗	96.3 ± 1.0

*The cell viability of the combination of rADI and 1 mM NMDA was different from the control and the 0.2 *μ*M (+)-MK-801 groups with values of *P* < 0.001 and *P* < 0.05, respectively.

**Table 2:** Vinyl-L-NIO triggered cytotoxicity in the presence of NMDA.

Cell viability	Control	Vinyl-L-NIO	Vinyl-L-NIO + NMDA
% of control	100 ± 3.5	98.4 ± 3.4	86.7 ± 1.2∗

*The combination of 5 *μ*M vinyl-L-NIO and 1 mM NMDA caused cytotoxicity compared to the control and vinyl-L-NIO alone groups with statistical significance of *P* < 0.05.
